# South African harm reduction guideline for chemsex

**DOI:** 10.4102/sajhivmed.v26i1.1763

**Published:** 2025-09-30

**Authors:** Andrew Scheibe, Yolaan Andrews, Ben Brown, Naeem Cassim, Thato Chidarikire, Johan Hugo, Regina Maithufi, Sive Mjindi, Dawie Nel, Shaun Shelly, Jabulile Sibeko, Mariette Slabbert, Londeka Xulu, Antons Mozalevskis

**Affiliations:** 1 TB HIV Care, Cape Town, South Africa; 2 Community Oriented Primary Care Research Unit, Department of Family Medicine, University of Pretoria, Pretoria, South Africa; 3 Population Health Sciences, Bristol Medical School, University of Bristol, Bristol, United Kingdom; 4 Networking HIV and AIDS Community of Southern Africa (NACOSA), Cape Town, South Africa; 5 Anova Health Institute, Cape Town, South Africa; 6 OUT LGBT Well-being, Pretoria, South Africa; 7 World Health Organization (WHO), Pretoria, South Africa; 8 National Department of Health, Pretoria, South Africa; 9 South African Network of People Who Use Drugs (SANPUD), Cape Town, South Africa; 10 South African National AIDS Council (SANAC), Pretoria, South Africa; 11 World Health Organization (WHO), Geneva, Switzerland, Switzerland

## Executive summary

The intentional use of psychoactive substances to enhance sexual experiences is known as chemsex. Chemsex is one form of sexualised substance use. Chemsex is primarily, but not exclusively, practised by key population groups including gay, bisexual and other men who have sex with men (GBMSM), transgender people, people who use drugs, and sex workers.

The potential harms result from the intersecting stigma and risks relating to substance use, criminalisation of drug use and possession for personal use, sex work, HIV and other sexually transmitted infections (STIs), prolonged and higher-risk sexual practices, substance-use disorders, mental health conditions, and sexual- and gender-based violence. Chemsex is not always problematic, but some people may develop health and/or social issues with this practice.

While data on the prevalence of chemsex in South Africa (SA) are limited, HIV and STI programmes for key populations regularly reach people who engage in chemsex. Chemsex sessions are frequently posted on GBMSM social networking and dating applications. This phenomenon is taking place in the context of increasing psychoactive substance use and a high prevalence of HIV and other STIs among key populations in the country.

Locally, there is a lack of knowledge, services, and support for people who engage in chemsex. This exacerbates their risk of exposure to HIV and other STIs, heightens barriers to accessing comprehensive care, and intensifies potential harms.

This guideline provides recommendations to address the key health and psychosocial aspects relating to chemsex in SA. Box 1 summarises the key components of chemsex harm reduction services. Recommendations are aligned with international evidence and informed by the professional experience of the authors, and research on the values and preferences of South African GBMSM who engage in chemsex.^1^ This guideline was thoroughly reviewed by external peer reviewers.

BOX 1 Key components of chemsex harm reduction services.

Services should be sensitive to sexual orientation, gender identity and expression, as well as substance use and sex work. Services should also:

- Employ a sex-positive approach.
- Provide judgement-free services that follow legal and ethical obligations.
- Enable accessible prevention, testing, treatment, harm reduction, and care services for HIV, sexually transmitted infections, viral hepatitis, and substance use.
- Promote and provide HIV pre-exposure prophylaxis (PrEP), post-exposure prophylaxis (PEP) and antiretroviral therapy (ART).
- Offer appropriate assessment, counselling, management, and referral for substance use and mental health needs, using a multidisciplinary approach.
- Facilitate access to emergency care, including for overdose, intimate partner violence, and sexual assault.

This guideline should be viewed within the context of the Southern African HIV Clinicians Guidelines for Harm Reduction.^2^

### Scope and purpose

- Provide an overview of chemsex in SA.
- Offer evidence-based clinical guidance for chemsex harm-reduction services.
- Provide a directory of useful resources and sensitised providers.

### Audience

This guideline is aimed at clinicians (doctors, nurses, and clinical associates); however, pharmacists, psychologists, social workers, programme officers, peer outreach workers, advocates, and policymakers may also benefit from the guidance provided. The term ‘healthcare provider’ has been used throughout and refers to all providers involved in providing health services for people who engage in chemsex.

### Guideline development

The authors made up the guideline development team. A subgroup of authors conducted a values and preferences research study among GBMSM engaging in chemsex in SA.^1^ This guideline is based on an international chemsex framework,^3^ that was adapted based on findings from the values and preference research,^1^ a scoping review of chemsex and harm reduction interventions, WHO guidance, and input from authors who provide services to people who engage in chemsex. The draft guideline was circulated for local and international peer review, and feedback and recommendations were integrated to produce a final version. Table 1 provides definitions of key terms.

**TABLE 1:** Definitions of key terms.

Term	Definition
Chemsex	The intentional use of psychoactive substances, including stimulant-, depressant-, and/or psychedelic-type substances, often in combination, in sexualised settings to enhance the sexual experience. Also known as party and play (PnP), High Fun, Chill, and Slam Sex (in the context of injecting drugs).^3,4^
Come-down	The process a person goes through when the effects of a drug wear off and brain chemistry returns to normal levels. It can last for a few hours to days, depending on the substance(s) taken and the dosage used. It can leave people feeling unhappy, anxious, or agitated. A come-down from methamphetamine use is also known as tweaking.^5^
Gay, bisexual, and other men who have sex with men (GBMSM)	This includes cisgender and transgender men who have sex with men, including both men who self-identify as gay and those who do not.^6^
Harm reduction	A comprehensive package of evidence-based interventions, based on public health and human rights, including needle–syringe programmes, opioid agonist maintenance therapy, and naloxone for overdose management.^7^ Harm reduction also refers to policies and strategies that aim to prevent major public and individual health harms, including HIV, viral hepatitis, and overdose, without necessarily stopping substance use.^8^ In this guideline harm reduction relates to interventions that address harms related to chemsex.
LGBTQIA+	An acronym for lesbian, gay, bisexual, transgender, queer, intersex, asexual, and additional identities and orientations not named. It is an umbrella term to describe a diverse spectrum of sexual orientations and gender identities that may be marginalised in society.^9^
Minority stress	The minority stress framework proposes that individuals from sexual minority groups (e.g. LGBTQIA+ people) experience more stress than heterosexual people do because of stigma, prejudice, and discrimination. The resultant stress can affect a person’s mental and physical health, and health behaviours, which in turn can lead to mental and physical disorders that are different to heterosexual people.^10,11^ Intra-minority stress refers to status-based competitive pressure GBMSM may face to compete with other men for social or sexual gain.^11^

Note: Please see full reference list of this article, https://doi.org/10.4102/sajhivmed.v26i1.1763, for more information.

## Introduction

Chemsex is defined as the intentional use of psychoactive substances (‘chems’) to initiate, facilitate, enhance and prolong sexual encounters.^12,13,14,15,16^ Chemsex is one form of sexualised substance use. In chemsex, stimulant, depressant and/or psychedelic-type substances are used, and often in combination.^16^ Other substances, such as amyl nitrate and medications for erectile dysfunction, may also be used.^16^ Substance use usually results in short-term euphoria, relaxation, increased sexual arousal, lowering of inhibitions, and a sense of emotional connection with sex partners.^1,17,18^ Chemsex can include an exploration of various sexual practices such as group sex, ‘marathon’ sex (sex lasting days) or other forms of sexual play (e.g. fetish and kink).^19,20,21^

The motivation to engage in chemsex is primarily to facilitate, sustain, and/or intensify sexual experiences and pleasure, and enhance connections among sexual partners.^18^ Among GBMSM and people from the lesbian, gay, bisexual, transgender, queer, intersex, asexual, and other identities (LGBTQIA+) community, it may also be linked to a short-term escape from internalised homo-/transphobia, internalised/self-stigma, low self-esteem, minority stress, intra-minority stress, or as part of transactional sex.^1,14,15,17,22,23,24,25,26^

In high-income settings, chemsex practices have been reported in up to a third of GBMSM.^16^ Available data suggest that chemsex among GBMSM from low- and middle-income countries (LMICs) in the past year ranges from 5.0% to 28.4%.^27^ Chemsex among transgender women and sexual minorities has been identified in several LMICs, but data are limited.^28^ For example, in Brazil, 40.7% of transgender women (*n* = 280) participating in a cross-sectional survey in 2020 reported chemsex in the past 6 months.^29^

The potential harms of chemsex relate to substance use (type of substance, dose, route of administration, and individual and contextual factors),^30^ unsafe sexual practices, increased exposure to HIV and other STIs, stigma, and the intersections between these components.^21,26^ The longer a chemsex session lasts, the higher the risk for blood-borne and sexually transmissible infections, psychosis and physical injury.^17^ Drug interactions between substances are often difficult to predict and pose significant risk, including overdose, loss of consciousness, and death.^17^ The psychoactive effects of substances may also affect the person’s ability to give informed consent.

Longer term consequences of ongoing chemsex include mood and anxiety disorders, and substance dependence.^3,31,32^ Prolonged engagement in chemsex may also lead to challenges in engaging in sex without the use of psychoactive substances.^17^ Stigmatisation can trigger social exclusion and restrict the ability of those who engage in chemsex to live authentically.^33^ These factors can negatively affect the mental health and wellbeing of people who engage in chemsex and are often barriers to accessing healthcare, psychosocial, and other services.^26^ There is also increased risk for violence.^1^ Additionally, the context in which chemsex takes place (e.g. in the context of sex work, at sex-on-premises venues or among people without stable housing), can exacerbate these potential harms.^16^ However, chemsex is not always harmful, nor problematic, and can contribute to social connection, sexual exploration, and deepened self-understanding.^18,21,32^

Distinguishing harmful from non-harmful substance use and identifying substance use disorders can enable triage and the provision of appropriate care. Harm reduction interventions should be offered to all people who engage in chemsex, despite moral objections (Box 2).^34,35^ People with harmful patterns of substance use or dependence should also have access to specialised services.^35^

BOX 2 Healthcare provider responsibility.

Healthcare providers should not pathologise chemsex participation based on moral judgement. Being registered as a healthcare provider confers one the right and privilege to practise a profession. Correspondingly, practitioners have moral and ethical duties to others and society in general. These duties are in keeping with the principles of the *Constitution of the Republic of South Africa*,^36^ and the obligations imposed on healthcare professionals by law.

Note: Please see full reference list of this article, https://doi.org/10.4102/sajhivmed.v26i1.1763, for more information.

### Chemsex in South Africa

Chemsex has emerged as a growing health concern in SA. Over the past 20 years, the availability and use of methamphetamine among adults in SA has increased dramatically; from < 0.5% reporting use in the past 3 months in 2002 to 1.5% in 2017.^37^

In 2012, over half (53.0%) of GBMSM and transgender women attending a sexual health clinic in Cape Town (*n* = 200) reported having ever had sex under the influence of substances, and 37.0% having ever used methamphetamine. In the same cohort, 30.0% reported group sex and 38.5% had engaged in transactional sex in the past year.^38^ More recent surveys^39,40^ among GBMSM and transgender women have identified frequent drug use, but did not explore chemsex practices. In 2019, the prevalence of methamphetamine use in the past 6 months among GBMSM in Cape Town and Johannesburg was estimated to range between 10.7% and 20.7%.^39^ In 2018/19, 40.2% – 66.6% of transgender women in three cities were estimated to have used drugs in the past 12 months.^40^

Programmatic and qualitative data from Cape Town and Johannesburg show that chemsex takes place in private residences, guest houses and sex-on-premises venues, and is facilitated through online platforms (e.g. Grindr®) and word of mouth.^1,25,26,41^

Psychoactive substances commonly used in chemsex are listed in Table 2. Crystal methamphetamine, known locally as ‘Tik’ or ‘Crystal’, is the most widely used substance, followed by crack cocaine. It is typically smoked but can also be injected (‘slamming’) or administered rectally (‘booty bumping’).^1,25,26,42^ The use of gamma-hydroxybutyrate (GHB)/gamma-butyrolactone (GBL) is reported to be more prevalent in affluent areas.^1^ Other substances commonly used in addition to chemsex drugs are outlined in Online Appendix 1, Table 2-A1.

**TABLE 2:** Psychoactive substances commonly used in the context of chemsex in South Africa.

Substance	Street names	Administration	Effects, benefits & half-life	Risks
Crystal methamphetamine	Meth, Crystal, Tik, Tina, Tjoef, Speed, Glass, Wubathala, Ice	Smoking, intranasal, intravenous injection, rectal	Stimulant Elevated mood Delayed ejaculation and prolonged orgasm Half-life: 4 h – 12 h	Come-down Insomnia Psychotic symptoms Hypertension Tachycardia Dependence Serotonin syndrome (increased risk if taken with other stimulants) Hallucinations (increased risk if taken with hallucinogens) Increased risk of cardiotoxicity if taken with GHB
Crack cocaine	Crack, Rocks, Ithse, Gatief, Akute, Letlpa, Litshe, Leswika	Smoking, intranasal, intravenous injection, rectal	Stimulant Elevated mood and energy Half-life: up to 1 h	Come-down Hypertension Mood disorder Dependence
Powder cocaine	Coke, Snarf, Puk, Sugar, Powder, Mfanya	Intranasal, buccal, intravenous injection	Stimulant Elevated mood and energy Half-life: up to 1 h	Come-down Hypertension Mood disorder Dependence
GHB/GBL	G, Gina, Liquid ecstasy	Oral, rectal (small doses 0.5 mL – 1.5 mL)	Stimulant (at lower doses) and depressant (at higher doses) Euphoria Sexual arousal Half-life: up to 7 h	Overdose (narrow range of doses for desired, non-toxic effects) Coma Dependence with severe withdrawal
Methcathinone	CAT, ephedrine	Smoking, oral, intranasal, intravenous injection	Stimulant Euphoria Sexual arousal Half-life: ± 1.5 h	Anxiety and paranoia Hypertension Seizures Dependence
Mandrax (methaqualone)	Ndanda, White pipe, buttons	Smoking (often combined with cannabis), oral	Depressant Sedative Half-life: 20 h – 45 h	Overdose (seizures, coma) Dependence

Note: Data were compiled from Cassim et al.^1^, Chemsex Toolkit^17^, Caldicott et al.^44^, Knudsen et al.^45^, Knudsen et al.^46^, Gable^47^, Methaqualone^48^, Grigg et al.^49^ and Williams et al.^50^; please see full reference list of this article, https://doi.org/10.4102/sajhivmed.v26i1.1763, for more information.

GHB, gamma-hydroxybutyrate; GBL, gamma-butyrolactone.

Condomless anal intercourse (‘barebacking’), sharing of injecting equipment, polysubstance use, and transactional sex occur locally in the context of chemsex.^41,43^ Research into the values and preferences of GBMSM who engage in chemsex identified preferences for services that provide accurate and non-judgemental information, enable informed decision-making and increase access to confidential and tailored physical and mental healthcare in safe spaces.^1^ Access to sterile injecting equipment, HIV pre-exposure prophylaxis (PrEP), STI testing and treatment, peer support, and post-violence care were also reported as priority needs.^1^

### Harm-reduction framework

**FIGURE 1:**
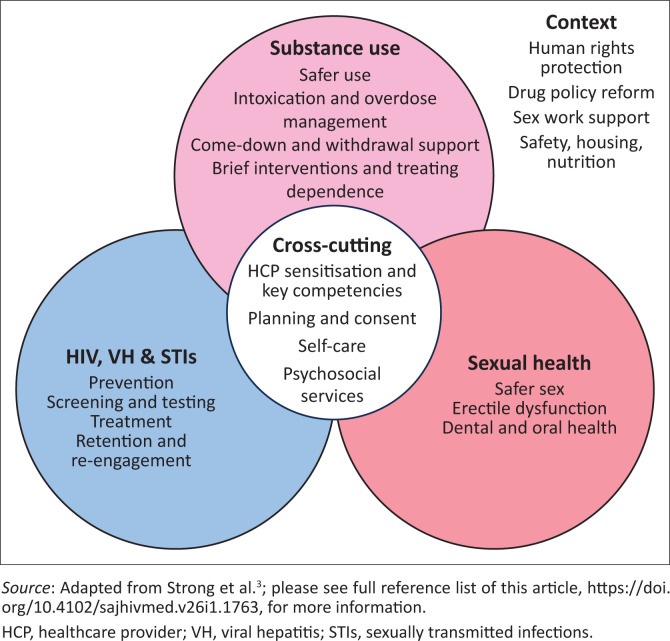
Framework for chemsex harm-reduction interventions.

Harm-reduction interventions in the context of chemsex can address issues relating to substance use, sexual health and STIs (Figure 1). This guideline provides an approach to clinical consultation and then provides additional information for the various components of the chemsex harm-reduction framework.

## Clinical consultation

The clinical consultation for a person who engages in chemsex should involve history taking, examination, diagnostic tests, and counselling and management plans that align to their unique risk profile and specific needs.^1,35,51^ A risk-reduction approach should be taken, and the consultation should take place in a safe space and be conducted in a non-judgmental manner. It is important to always explain why an examination is being done and what it will entail.

### History, examination and diagnostics

Table 3 details what should be assessed during a clinical consultation. A more detailed history should be taken at the initial visit. Changes should be enquired about in subsequent (3–6-monthly) visits.

**TABLE 3:** Components of the clinical consultation.

Assessment		Details
History	Demographics	Preferred name and pronouns, age, gender, place of residence, occupation/income source
	Medical history†	Medical conditions, including HIV, viral hepatitis, previous STIs, erectile disorder and other chronic conditions (e.g. hypertension, diabetes, ischaemic heart disease, prostate cancer) Medications, including HIV PrEP, HIV PEP, doxyPEP, ART, erection medications Allergies
	Social history†	Partner status Family, friends, social support
	Sexual history (including chemsex)	Context of chemsex, including locations, frequency, duration, partners Substances used during chemsex, including types, method of administration, mixing and duration of use ■ Psychological experiences of come-down ■ Previous chemsex-related adverse events: loss of consciousness, substance-induced psychosis Sexual behaviour: ■ Type of intercourse and position (e.g. anal intercourse, sexual positioning [insertive/receptive/versatile intercourse]) ■ Other forms of play (e.g. fisting) ■ Condom and lubricant use ■ Existence of erectile dysfunction, difficulty with ejaculation and other sexual dysfunction (e.g. pain) ■ Sexual behaviour without substances Sexual violence or constraint, and non-respect for consent
	Mental health†	History of mental health conditions, including psychotic episodes and post-traumatic stress disorder Screen for common conditions (anxiety, depression, psychosis, suicidal ideation), using a validated screening tool (see useful screening tools in Online Appendix 1, Table 1-A1). Past sexual abuse or other traumas History of sexual addiction Client’s view of their identity, sexuality, sexual practices and chemsex Client’s view about wanting to change any practices or behaviours linked to chemsex
	Substance use	Alcohol and substance use assessment outside of chemsex (see useful screening tools in Online Appendix 1, Table 1-A1).
	Symptom screen	General STIs Tuberculosis Substance-related
Examination	Vital signs	Pulse, blood pressure, respiratory rate, SpO_2_
	Oral	Dental hygiene, ulcers
	Skin	Injection marks, formication, thrombophlebitis
	Cardiovascular	Assess for infective endocarditis if signs of injecting
	Anogenital	Trauma, discharge, ulcers
	Substance use and signs of withdrawal	Signs of stimulant intoxication (jittery, tachycardia, hypertension, sweating, hyperthermia, dilated pupils, psychosis‡) or withdrawal (dysphoria, irritability, fatigue, insomnia or (more commonly) hypersomnia, psychomotor agitation or retardation) Signs of depressant use (calm or depressed mood, reduced level of consciousness, small pupils, bradycardia) or withdrawal (fatigue, tremors, sweating, pupil dilation).
Diagnostics	Rapid test	HIV§ Syphilis§ HBV¶ and HCV††
	Laboratory testing	For other STIs and to inform treatment‡‡
	Work-up	HIV, STI and viral hepatitis work-up, as needed
Management	Counselling, prevention and treatment services, and referral as needed (see section, Management)	

Note: For more details, please see Peters et al.^52^, Peters et al.^53^ and National Department of Health (NDoH)^54^; please also see full reference list of this article, https://doi.org/10.4102/sajhivmed.v26i1.1763, for more information.

ART, antiretroviral therapy; STIs, sexually transmitted infections; PrEP, pre-exposure prophylaxis; PEP, post-exposure prophylaxis; SpO_2_, peripheral capillary oxygen saturation; HBV, hepatitis B virus; HCV, hepatitis C virus; HSV, herpes simplex virus.

† , Ensure thorough history at first visit then assess for changes at consequent visits;

‡ , Symptoms may include auditory or visual hallucinations, paranoia and delusional thoughts;

§ , Combined HIV and syphilis rapid tests can also be used;

¶ , Do Hepatitis B surface antigen testing at first visit only then offer vaccination/treatment;

†† , Hepatitis C virus molecular testing is required for people with a reactive hepatitis C antibody test;

‡‡ , Where resources allow, asymptomatic molecular testing should be offered 3-monthly for *Chlamydia trachomatis* and *Neisseria gonorrhoea*. Molecular testing for HSV and syphilis may be considered in the presence of a genital ulcer to inform treatment.

### Management

It is important to provide the client with an individualised management plan. Consider the following:

- Vaccination:
  ■ Consider hepatitis A, hepatitis B, human papilloma virus (HPV) and mpox vaccination.
  ■ See the National Department of Health (NDoH) viral hepatitis guidelines^54^ and NDoH mpox guidance^55^ for more detail.
- Information, Education and Communication (IEC):
  ■ Provide accurate information on substances, safer substance use, drug-drug and drug-medication interactions, safer sex and sexual reproductive health, and mental health.
- Counselling:
  ■ Discuss risk-reduction (including on overdose) and safety management plan (including crisis support).
  ■ Provide additional information on other specific health condition(s) as needed.
  ■ If not living with HIV, explain post-exposure prophylaxis (PEP) and encourage PrEP. See the latest NDoH PEP guideline, Southern African HIV Clinicians Society (SAHCS) PEP guideline, NDoH PrEP guideline and SAHCS PrEP guideline for more detail.^56,57,58,59^ If available, offer long-acting injectable PrEP.
  ■ If living with HIV, explain undetectable = untransmissible (U = U) and support adherence.
  ■ Ask about intimate and family relationships, and self-care.
  ■ Provide condoms and lubricants.
- Provision of doxyPEP^60^:
  ■ Discuss the risks of syphilis, chlamydia and gonorrhoea infection, and the pros and cons of doxyPEP.
  ■ Following shared decision-making, provide a prescription for the self-administration of doxycycline 200 mg orally as soon as possible and within 72 h after having oral, vaginal or anal sex (maximum dose is 200 mg every 24 h). Prescribe sufficient doses based on the client’s planned sexual activity until their next visit. Reassess the need for doxyPEP every 3–6 months.
- Referral:
  ■ Provide information on where to access harm reduction initiatives, specialised drug treatment services, mental health services and psychosocial support. See Online Appendix 1, Table 1-A1 for available resources.

People who engage in long-term, intense chemsex may benefit from long-term support to resolve their substance use issues and gain control of their sex lives. Considerations of interventions should take the person’s life stage into account (see Box 3).

BOX 3 Special considerations for younger people and older people.

**Younger people:**

- Young people may be at greater risk of harm because of the potential impact of substances on neuro-cognitive development.
- Dependence and blood-borne infection risk is greater in younger people because of potentially longer-term substance use and engagement in transactional chemsex sessions.
- Young people may have increased vulnerability to sexual assault, coercion, and exploitation.
- Young people require tailored counselling and mental and healthcare services to address:
  ■ Underlying issues contributing to vulnerability, substance use, and unsafe sexual behaviour.
  ■ Support to help reduce or stop harmful drug and/or alcohol use.
  ■ Peer support with other young people who engage in chemsex, to share experiences, seek advice, and access resources.

**Older people:**

- Healthcare providers may not enquire about substance use in older clients.
- Symptoms of substance use may be masked by age-related cognitive or physical changes.
- There is an increased risk of drug-drug interactions in older men as they are more likely to be on chronic medication. Some substances may also cause or exacerbate erectile dysfunction. The use of medications for erectile dysfunction should be specifically asked about.
- Reasons for engagement in chemsex may be unique, including issues relating to aging, identity, isolation and desire to engage with younger sex partners.
- Older men who engage in chemsex may also require support around nutrition and self-care and link to relevant services for older GBMSM.

*Source:* Adapted from Irfan et al.^61^, TheBody^62^, Addiction(s): recherches et pratiques [Addiction(s): Research and practices]^63^; please see full reference list of this article, https://doi.org/10.4102/sajhivmed.v26i1.1763, for more information.

GBMSM, gay, bisexual and other men who have sex with men.

### Retention and re-engagement in care

Adherence support should be provided to increase the clients’ retention in care. It should focus on supporting persistence on PrEP or ART, as appropriate. Counselling should support people around maximising the protective effect of PrEP or of HIV viral suppression (including clear messaging around U = U), respectively. People who have experienced disruptions in care should be supported to re-engage in care.

## Substance use

### Supporting safer substance use

Selected interventions to reduce substance-related harms are included in Table 4 and are detailed in the SAHCS harm reduction guidelines.^2^

**TABLE 4:** Supporting safer substance use.

Intervention	Note
Safer drug administration	Encourage people who inject drugs to consider smoking or snorting drugs to reduce potential harm.
Safer injecting	Provide accurate information on clean procedure, recommended veins for injecting (and areas to avoid) and direction of injecting. Counsel about the need to have sufficient sterile needles and syringes based on their injecting practice and advise on where to access these. Encourage the use of low dead-space needles and syringes. Counsel on the risks of sharing equipment and methods to reduce risks of infection. Encourage colour-coding or other ways of uniquely identifying needles for identification of a person’s equipment.
Minimising drug-drug and drug-medication interactions	Substances that interact with methamphetamine are listed at https://go.drugbank.com/drugs/DB01577
Safer drug dosing^64^	Encourage clients to familiarise themselves with the effects of substances in a safe environment and with another trusted person, before using it in a chemsex situation. Suggest that the client uses written notes during a chemsex session with drug timing and pre-measured doses. If using GHB, encourage syringes for accurate measurement and to ‘start low and go slow’. Dosing is affected by the purity and concentration of liquid GHB and tolerance.†
Safer smoking^17,65^	Explain that pathogens may be transmitted through saliva or blood when sharing pipes and that home-made smoking equipment may release toxic fumes. Encourage the use of glass stems and pipes of appropriate length to prevent burns. Plastic mouth pieces and the use of aluminium foil can also reduce burns. Encourage the use of lip balm, chewing gum and suckers.
Safer nasal ingestion (snorting)^64^	Explain that crushing substances before insufflation, alternating nostrils, use of a clear surface to inhale off, irrigation of nostrils with saline solution and the use of a tube can prevent damage to the nasal mucosa. Encourage the use of disposable and flexible tubes (these can be individualised using different colours). Encourage use of a clean plastic card, small cosmetic scoop or plastic razor blade for handling drugs.
Safer rectal administration (booty bumping)^66^	Explain that the harms of drug insertion into the anus can be reduced by mixing the drug(s) with water and drawing it up into a syringe, removing the needle and then inserting it into the anus. Encourage the use of lubricant on the syringe and inside of the anus to reduce trauma. Suggest spacing out periods of rectal administration with other routes of administration to allow time for anal tissue to heal. Encourage thorough cleaning of equipment used.
Drug-checking services	The chemical analysis of drug samples and provision of these results to people who use drugs. This harm reduction intervention is not widely available in South Africa.

*Source:* Adapted from Scheibe et al.^2^, World Health Organization^35^, United Nations Office on Drugs and Crime^67^ and Maghsoudi et al.^68^; please see full reference list of this article for more information.

Note: Please see full reference list of this article for more information.

GHB, gamma-hydroxybutyrate; GBL, gamma-butyrolactone.

† , GBL is converted to GHB in the body. A typical initial dose in a European setting is about 0.8 mL – 1.2 mL of GBL or 1 mL – 2 mL of GHB.^69^

### Intoxication and overdose management

Intoxication refers to a transient condition following the intake of a psychoactive substance resulting in disturbances of behaviour, perception, cognition, affect, perception and/or consciousness.^70^ Prevention involves educating clients on risks, risk reduction and detection of overdose and emergency management. Clients should be informed about potential bulking agents and contaminants in illicit substances, which could include a range of new psychoactive substances and other adulterants.^17^

#### Stimulants

A stimulant overdose is characterised by hyperthermia while being conscious. Management involves the use of long-acting benzodiazepines (e.g. a titrated dose of 5 mg – 10 mg diazepam orally, intravenously, or per rectum). Antipsychotic medication may be needed as an adjunct (e.g. haloperidol, 1 mg – 2.5 mg orally or intramuscularly). Additional supportive therapy is required to manage people with chest pain, tachyarrhythmias or additional neurological symptoms. Following an episode of stimulant intoxication, clients should be screened for suicidality and managed accordingly.^70^

#### Gamma-hydroxybutyrate/gamma-butyrolactone

High doses (1.25 mL – 2 mL) of GHB^71^ can lead to central nervous system and respiratory depression, and potentially death. Management is supportive, with airway protection and mechanical ventilation if needed. Naloxone may be given to exclude co-ingestion of an opioid, though it is not effective for reversing the effects of GHB/GBL.

#### Opioids

Chemsex does not usually involve opioids (depressants), but opioids may be used concomitantly and, in some cases, unintentionally (e.g. consuming stimulants contaminated by fentanyl). Management involves respiratory support and administration of naloxone. Additional information on the management of overdose is detailed in the SAHCS Harm Reduction guidelines.^2^

### Come-down and withdrawal support

A come-down is the physical and psychological after-effects of substance use, often characterised by fatigue, mood swings, anxiety, low mood and physical discomfort. Clients who have developed tolerance for or dependence on substances may experience a withdrawal syndrome after a period of abstinence. Managing a come-down safely is crucial to reduce harm and support recovery. Guidance on come-down support is included in Online Appendix 6, Table 1-A1.

#### Methamphetamine

Management should focus on supportive and symptomatic treatment (e.g. anti-emetics for nausea, simple analgesics for pain, light sedatives for insomnia). Depressive symptoms may occur, and healthcare providers should be alert for and screen for the risk of suicide.

#### Gamma-hydroxybutyrate/gamma-butyrolactone

The come-down from GHB/GBL can involve physical and psychological symptoms such as fatigue, anxiety, depression, and in some cases, withdrawal symptoms.^14,16^ Withdrawal is characterised by anxiety, agitation, tremors, insomnia, sweating and an increased heart rate. In severe withdrawal hallucinations, confusion, seizures, and delirium may occur, which can be life-threatening.^14^ Urgent medical attention is required in these cases. Management involves the use of benzodiazepines tailored to the individual’s symptoms and hospitalisation may be required.^72^

### Brief interventions and treating dependence

A non-judgemental, harm reduction approach should be used when engaging clients around changes in drug use. Interventions could include motivational interviewing screening brief intervention and referral to treatment, cognitive behavioural therapy, and contingency management.^13,16,73,74,75^ Additional guidance on substance use interventions are included in the SAHCS harm reduction guidelines.^2^

## Sexual health

Sexual health interventions include encouraging safer sex, managing erectile dysfunction, and encouraging good dental and oral health.

### Safer sex

Information on safer sexual practices with a lower risk of transmission (e.g. oral sex, mutual masturbation) and methods to reduce the risks of HIV and STI transmission during anal sex should be provided. Examples include:

■ If ejaculation in the mouth occurs, spit out the semen and rinse mouth with water.
■ Avoid oral sex in the presence of open wounds or bleeding in the mouth.
■ Use condoms with compatible lubricant for anal intercourse.
■ Reduce the number of sexual partners, or those with whom sex is unprotected.
■ Avoid ejaculation in the anus by removal of penis before ejaculation.
■ Avoid engaging in sexual practices that may cause anal lesions (e.g. fisting).
■ Minimise the use of laxatives as these weaken the anal mucosa.
■ Avoid sharing of sex toys.
■ Avoid sharing of lubricant (write initials on bottle) and have individual towels or use disposable towels.
■ Use latex gloves with water- or silicone-based lubricant.

### Erectile dysfunction

GBMSM engaging in chemsex may experience erectile dysfunction, either because of prolonged sex or as a drug side effect. It is important to exclude chronic conditions and medications as causes by taking a thorough medical history. Treatment options should be discussed, including the use of a phosphodiesterase-type 5 inhibitor such as sildenafil. If a client is started on sildenafil, or other erectile dysfunction treatment medications, it is important to educate them about possible medication reactions that may cause hypotension. This includes ritonavir (maximum sildenafil dose 25 mg in 48 h), amyl nitrite (poppers) and cobicistat.

### Dental and oral health

Encourage clients to maintain regular brushing and to take toothpaste, a toothbrush and mouthwash to chemsex sessions. Chewing gum can also be helpful for a dry mouth.

## Cross-cutting interventions

Services for people who engage in chemsex should aim to address the intersecting risks related to substance use and sexual behaviour (Figure 1). The context in which these occur is also important. Guidance on implementing interventions that are tailored to the context of chemsex are outlined below.

### Healthcare provider sensitisation and key competencies

Sensitisation of healthcare providers should include the following topics:

- Sexual orientation, gender identity and gender expression
- Transactional sex and sex work
- Drug classes, effects, risks and potential interactions
- Harm reduction, treatment interventions and referral options.

Key competencies for healthcare providers providing chemsex-related services include:

- Effective communication and ability to develop a trusting therapeutic relationship
- Knowledgeable about priority health issues relating to chemsex
- Appropriate history taking around sexuality, sexual behaviour, and drug use
- Effective counselling and engagement techniques (e.g. motivational interviewing)
- Use of appropriate screening and diagnostic tools
- Assessment triage and assessment of risks
- Joint decision-making and health management planning aligned to the client’s personal health goals.^17^

### Planning and consent

Healthcare providers should be aware and acknowledge that a client’s ability to develop or implement harm reduction plans may be affected by substance dependence and/or a desire for chemsex. Clients should be supported to think through feasible plans to enable safer chemsex sessions. Healthcare providers should emphasise that participation in chemsex sessions does not mean that a person loses their right to choose, and control their body and sexual experiences.^76^

Clients should be reminded that the ability to provide consent can be affected by substance use. Clients should be encouraged to agree with their sexual partner on acceptable sexual behaviour prior to a chemsex session. Discussions around consent should also include the taking of photos or videos.^77^ It is important to remind clients that it is against the law to have sex with someone who is unconscious or asleep, which could result in sexual assault charges.^76^

### Self-care

Self-care is the ability of individuals, families and communities to promote and maintain their own health, prevent disease, and to cope with illness.^78^ Self-care can be encouraged by healthcare providers and peers, and services can be provided in-person or online. These may include:^35,78,79^

- Self-assessment questionnaires around wellbeing (e.g. Self-care-inventor^80^), drug use (e.g. WHO eASSIST^81^) and mental health conditions (e.g. for anxiety^82^)
- IEC materials to help clients to increase personal responsibility to reduce harms
- Self-collection of samples for STI testing, including self-testing for HIV and syphilis^83^ (and hepatitis C, once locally available)^35,84^
- ‘Sign posting’ and guiding people on how to join peer and other support groups (see Online Appendix 1, Table 1-A1), and where to access nutrition, safe housing, mental health services and other psychosocial support.

### Psychosocial services

Stigma surrounding chemsex is complex, intersecting with internalised stigma and societal views on substance use, sexuality and health, and occurs at multiple levels.^14,16,17,73,85^ Stigma can result in mental health challenges, social exclusion, barriers to health and social services, and unsafe sex behaviours.^14,16,17,73,85^ Psychosocial services should be provided by trained and sensitised healthcare providers. Psychological assessment, counselling and therapy, trauma-informed care, post-violence care, crisis intervention, facilitated disclosure, and family therapy may provide benefit to clients.

## Conclusion

Chemsex is a contemporary phenomenon and is becoming more common in SA. Healthcare providers should be sensitised to chemsex and be capacitated to provide supportive and evidence-based care to people who engage in chemsex. The use of a harm reduction approach that addresses risks related to substance use and sexual practices can reduce potential harms and support people to engage in safer chemsex. Online Appendix 1 includes a range of useful tools.
